# An Endogenous Proton-Powered Adaptive Nanomotor for Treating Muscle Atrophy

**DOI:** 10.3390/ma18061351

**Published:** 2025-03-19

**Authors:** Ming Liu, Zhicun Liu, Xiangkai Qiao, Cheng Chen, Hongtu Guo, Hao Gu, Junbo Li, Tiedong Sun

**Affiliations:** College of Chemistry, Chemical Engineering and Resource Utilization, Northeast Forestry University, Harbin 150040, China; 6ming@nefu.edu.cn (M.L.); 2022214670@nefu.edu.cn (Z.L.); guoht@nefu.edu.cn (H.G.)

**Keywords:** biocatalytic, nanomotor, proton gradient, negative chemotaxis, muscle atrophy

## Abstract

Nanomotors driven by endogenous enzymes are favored in biology and pharmacy due to their spontaneous driving and efficient biocatalytic activity, and have potential applications in the treatment of clinical diseases that are highly dependent on targeted effects. For diseases such as muscle atrophy, using energy molecules such as ATP to improve cellular metabolism is a relatively efficient treatment method. However, traditional adenosine triphosphate (ATP) therapies for muscle atrophy face limitations due to instability under physiological conditions and poor targeting efficiency. To address these challenges, we developed an endogenous proton-gradient-driven ATP transport motor (ATM), a nanomotor integrating chloroplast-derived F_o_F_1_-ATPase with a biocompatible flask-shaped organic shell (FOS). The ATM is synthesized by vacuum-injecting phospholipid-embedded F_o_F_1_-ATPase nanothylakoids into ribose-based FOS, enabling autonomous propulsion in acidic microenvironments through proton-driven negative chemotaxis (directional movement away from regions of higher proton concentration). This nanomotor converts proton gradients into ATP synthesis, directly replenishing cellular energy deficits in atrophic tissues. In vitro studies demonstrated high biocompatibility (>90% cell viability at 150 μg/mL) and pH-responsive motility, achieving speeds up to 4.32 μm/s under physiological gradients (ΔpH = 3). In vivo experiments using dexamethasone-induced muscle atrophy mice revealed that ATM treatment accelerated weight recovery and restored normal muscle morphology, with treated mice exhibiting cell sizes comparable to healthy controls (30–40 μm vs. 15–25 μm in untreated). These results highlight the ATM’s potential as a precision therapeutic platform for metabolic disorders, leveraging the natural enzyme functionality and synthetic material design to enhance efficacy while minimizing systemic toxicity.

## 1. Introduction

Atrophy is defined as a reduction in the size of a tissue or organ, which occurs as a result of cell contraction. Specifically, the decrease in cell size is attributed to the loss of organelles, cytoplasm, and proteins [1]. On the other hand, muscle wasting pertains to the loss of muscle mass and strength, which can be caused by illness [2], malnutrition, a lack of exercise, or the aging process [3]. Given the multifactorial etiology of muscle atrophy, therapeutic approaches target distinct pathophysiological pathways, including medication [4], physical therapy [5], nutritional support [6], and electrical stimulation therapy [7]. The utilization of ATP-based therapeutics in treating muscle atrophy is predominantly founded on their functions in energy metabolism and muscle function. For instance, ATP analogs can directly supply energy, which promotes the metabolic activity of muscle cells, enhancing muscle function and strength [8]. Moreover, they can also foster muscle protein synthesis and decelerate the progression of muscle atrophy. This is achieved by activating specific signaling pathways, such as the mammalian target of rapamycin (mTOR) pathway [9]. In addition, ATP compounds might facilitate the recovery of muscle function. They mediate this effect via dilating blood vessels and enhancing microcirculation, thus increasing the supply of oxygen and nutrients to muscle tissue [10,11,12].

Nanomotors represent a category of minuscule, self-propelled nanostructures that possess the ability to move autonomously within a microscopic liquid milieu [13]. Owing to their distinctive locomotive capabilities and maneuverability, they have demonstrated a broad spectrum of therapeutic applications in the medical domain [14]. Specifically, some nanomotors can be engineered to carry drugs and precisely transport them to specific diseased tissues or cells [15], which not only enhances the therapeutic efficacy of the drug but also mitigates side effects. Notably, in cancer treatment, nanomotors can directly deliver chemotherapy drugs to the tumor site, thereby targeting tumor cells while avoiding damage to the surrounding healthy cells [16]. For instance, they are capable of crossing the blood–brain barrier and delivering chemotherapy drugs directly to brain tumor cells [17]. In addition, they can traverse the body, gather information regarding disease states, or directly assay biomarkers within the body [18].

Protein molecule motors in living organisms operate efficiently under normal physiological conditions [19,20,21]. Consequently, we can capitalize on the characteristics of the rotational protein molecule F_o_F_1_-ATPase. This enzyme can utilize adenosine diphosphate (ADP) and inorganic phosphate (Pi) groups to synthesize ATP under the influence of the proton-gradient potential. Notably, the integration of chloroplast-derived nanothylakoids into liposomal systems represents a biomimetic strategy to preserve the structural integrity and catalytic activity of F_o_F_1_-ATPase [22]. The nanothylakoid membrane, as a natural photosynthetic machinery, inherently contains densely packed F_o_F_1_-ATP synthase complexes that exhibit remarkable proton-gradient-driven ATP synthesis efficiency [23]. By encapsulating these nanothylakoids within phospholipid bilayers, enhanced stability against enzymatic degradation while maintaining their capacity for directional proton-driven transport can be achieved [22]. This approach leverages the evolutionary optimization of plant-derived energy conversion systems, providing a sustainable and scalable platform for ATP production in therapeutic applications. This enables their utilization for the efficient treatment of muscle dystrophy diseases. Furthermore, the microenvironment of muscular dystrophic tissues exhibits significant acidosis (pH~6.5–6.8) due to impaired mitochondrial oxidative phosphorylation and lactate accumulation [24,25,26]. This pathological acidosis arises from two synergistic mechanisms: (1) the reduced ATP synthase efficiency leading to compensatory glycolytic flux [27], and (2) the defective monocarboxylate transporter 4 (MCT4)-mediated lactate export in atrophic myofibers [28]. Thus, this F_o_F_1_-ATPase-type motor holds great promise for application in the treatment of such metabolic diseases.

**Scheme 1:**
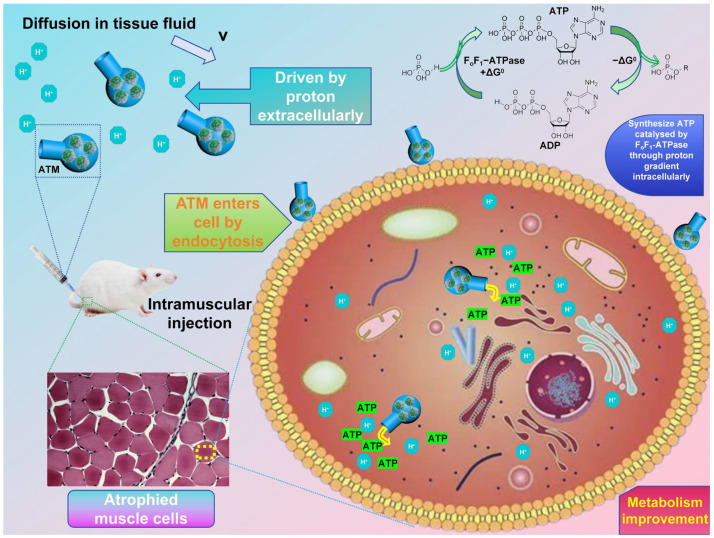
Mechanism of action of endogenous proton-driven nanomotor ATM for the treatment of muscular dystrophy.

In this study, based on the research status in related fields, we developed a highly biocompatible F_o_F_1_-ATPase-powered system (Scheme 1). This system, which is named the ATP transport motor (ATM), is equipped with a lecithin-assembled spontaneously-driven nanoscale engine and has a round-bottom flask-like shell. It is assembled through the vacuum injection of the protein lipid engine (PLE) embedded with F_o_F_1_-ATPase and a flask-shaped shell (FOS). The FOS is synthesized via a hydrothermal method, using pentose as the raw material. When a proton concentration gradient is present, the ATM can be moved by the rotation of F_o_F_1_-ATPase. This rotation drives the motor to swim negatively in the direction of decreasing proton concentration. At the same time, as protons enter the ATM through its opening, they will drive the internal F_o_F_1_-ATPase to use the ADP and phosphate groups to synthesize and store ATP. By testing the cell activity of this motor-based drug, it becomes evident that it exhibits good biocompatibility. Thus, it holds great promise and potential for experimental animal treatment research. Given the properties of this drug motor, we investigated its efficacy in treating muscular wasting symptoms induced by dexamethasone. The experiment demonstrated that mice treated with ATM exhibited faster average weight gain compared to untreated mice, approaching the weight of healthy controls. Through the observation of muscle tissue morphology, it was found that ATM indeed improved the symptoms of muscle atrophy, causing the cell morphology of the diseased tissue to be more similar to that of normal cells. The treatment with ATM offers a practical and promising approach for atrophic diseases. Due to its high biocompatibility and minimal side effects, it is anticipated to be translated into clinical applications in the future.

## 2. Materials and Methods

### 2.1. Synthesis of Protein Lipid Engine (PLE)

According to previous works of research [22] and related improvement plans, after washing the fresh spinach leaves, dry and remove the petiole and main vein. Then, 10 g of the leaves were weighed, cut, and placed in a mortar for further crushing. Subsequently, 40 mL of chloroplast buffer (comprising 0.4 M sucrose (C_12_H_22_O_11_, biotechnology level, Macklin, Shanghai, China), 0.03 M KH_2_PO_3_ (suitable for molecular biology, ≥99%, Aladdin, Shanghai, China), 0.01 M Na_2_HPO_4_ (anhydrous grade, used for drug research, EP, BP, JP, USP, Macklin, Shanghai, China), 0.01 M KCl (molecular biology specific, 99.5%, Macklin, Shanghai, China), and 2 mM vitamin C (C_6_H_8_O_6_, >99.0% (T), Macklin, Shanghai, China)) was poured in and stirred well. The mixture was filtered through eight layers of gauze, and the homogenate was collected. The homogenate was centrifuged at 500 r/min for 10 min before the supernatant was collected. Next, 2 mL of 50% (*w*/*w*) and 15% (*w*/*w*) sucrose solutions were added to the centrifuge tube in sequence to establish a discontinuous density gradient. The 50% sucrose layer (density ~1.23 g/cm^3^) provides sufficient buoyant force to retain intact chloroplasts while allowing heavier cellular debris to sediment, whereas the 15% sucrose layer (density ~1.06 g/cm^3^) facilitates the separation of chloroplasts from lighter cytoplasmic components [29]. It should be noted that the 15% (*w*/*w*) sucrose solution was slowly injected along the centrifuge tube wall, and the surface of the 50% (*w*/*w*) sucrose solution should not be stirred. Then, 2 mL of the supernatant was slowly added along the tube wall on this density gradient. After that, density-gradient centrifugation was carried out again at 8000 r/min. The chloroplasts were aspirated from the intermediate layer using a dropper, washed with the above-described buffer, and centrifuged. After being suspended in a solution containing 10 mM HEPES (C_8_H_18_N_2_O_4_S, suitable for molecular biology, ≥99.5% (T), Aladdin, Shanghai, China)-KOH (≥95%, Aladdin, Shanghai, China) and 5 mM MgCl_2_ (anhydrous, 99%, Macklin, Shanghai, China) at pH 7.8, they were placed in a dark refrigerator freezer for 20 min to allow the chloroplasts to dissolve. The resulting suspension was centrifuged at 8000 r/min for 10 min. The obtained nanothylakoid pellets were collected and resuspended in a solution containing 50 mM HEPES-KOH, 100 mM sorbitol (C_6_H_14_O_6_, ≥98%, used for drug research, Macklin, Shanghai, China), and 10 mM MgCl_2_ at pH 7.5. Finally, it was mixed with an equal mass of lecithin (C_42_H_80_NO_8_P, from soybean, >98%, Macklin, Shanghai, China) liposomes (containing phosphate, choline, fatty acids, glycerol, glycolipids, triglycerides, and phospholipids) and stirred well to obtain a light-green suspension. This suspension was stored in a refrigerator at a temperature of 4 °C and preserved as PLE.

The TEM image of PLE was obtained using a transmission electron microscope model 13004511 JEM-2100 (200 kV) (Tokyo, Japan). Centrifuge PLE and disperse it using the same buffer solution, dilute it to the appropriate concentration, and drop it onto a carrier to wait for drying. When loading the sample, place the carrier net on the sample rod and check for looseness. Then locate the sample rod on the angle measuring table and start pre-vacuuming. After vacuuming, rotate the sample rod according to a certain method to allow it to enter the tube. Check the status of the electron microscope, and eliminate the astigmatism in the aperture of the condenser lens. Adjust the current state, center the Beam shift, ensure that Beam Tilt PPX coincides with Beam Tilt PPY, and then calibrate the rotation center. Open the Beam and select the observation area, and then move to the center of the field of view. Select a scale bar of 500 nm to obtain a TEM image of PLE. Then, using the ImageJ (TrackMate v6.0.1) software for analysis and processing, the particle size distribution statistics of PLE can be obtained. Set the scale according to the reference size in the TEM image of PLE on ImageJ, draw line segments along the diameter of each particle in the image, and calculate the size distribution of all particles. Then, use Origin 2024 (10.1) software to select the minimum distribution interval of 50 nm, import all particle sizes, and count them according to the numerical distribution to obtain the particle size statistical graph.

### 2.2. Synthesis of Flask-Shaped Organic Shell (FOS)

FOS was synthesized following the procedures described in the previously published research [30,31] and methods from self-exploration. Firstly, 0.0435 g of P123 (Poly (ethylene oxide)-poly (propylene oxide)-poly (ethylene oxide) triblock copolymer, H(OCH_2_CH_2_)_x_(OCH_2_CHCH_3_)_y_(OCH_2_CH_2_)_z_OH, average Mn ~5800, Macklin, Shanghai, China), and 0.0365 g of sodium oleate (C_18_H_33_NaO_2_, 98.0%, Macklin, Shanghai, China) were added to 20 mL of deionized water. The selection of P123 as a structure-directing agent and sodium oleate as co-template was based on their synergistic effects in controlling the hierarchical assembly process. While P123 provides the primary micellar template for ribose polymerization through its amphiphilic block structure, sodium oleate enhances the structural stability through hydrophobic interactions with the poly (propylene oxide) core [31]. The residual template molecules were effectively removed by centrifugal washing after the hydrothermal process, minimizing potential immunogenicity. The mixture was stirred gently to form a clear solution. Subsequently, it was combined with a 40 mL aqueous solution containing 3 g of ribose (C_5_H_10_O_5_, ≥99%, Aladdin, Shanghai, China). The resulting mixture was stirred at room temperature for 30 min to obtain a clear solution. Afterward, it was transferred to a 100 mL hydrothermal reactor, being placed in an oven and hydrothermally treated at 160 °C for 12 h. Once the treatment was complete, the reactor was allowed to cool to room temperature. The black solid was then retrieved by centrifugation, washed with deionized water, and subsequently dried in an oven at 60 °C, thus obtaining FOS solid particles.

### 2.3. Synthesis of ATP Transport Motor (ATM)

In accordance with the methods from relevant literature [22] and corresponding pre-experimental results, 5 mg of FOS pellets are incorporated into 10 mL of an ADP buffer. This buffer contains 90 mg of PLE liposomes, which is formulated with 10 mM Tricine (C_6_H_13_NO_5_, used for cell culture, ≥99.5% (T), Aladdin, Shanghai, China)-NaOH (≥99%, Aladdin, Shanghai, China), 20 μM ADP (C_10_H_15_N_5_O_10_P_2_, ≥95%, Aladdin, Shanghai, China), 5 mM NaH_2_PO_4_ (suitable for molecular biology, ≥99%, Aladdin, Shanghai, China), 2.5 mM MgCl_2_, and 30 mM NaCl (suitable for molecular biology, ≥99.5% (AT), Aladdin, Shanghai, China), at a pH of 7.8. Subsequently, using a vacuum pump, the solution is pumped to a pressure of −0.1 bar (gauge pressure) for around 30 min. After the vacuum-treatment step, the solution was transferred to a 0 °C environment and underwent bath sonication (40 kHz frequency, 100 W power, JY92-IIN ultrasonic homogenizer, Ningbo Scientz Biotechnology Co., Ltd., Ningbo, China) for 1 h.

The SEM image of ATM was obtained using a scanning electron microscope model TESCAN MIRA LMS from Brno, Czech Republic (10 kV). Centrifuge the buffer solution containing ATM to obtain ATM particles, dry them, disperse them on conductive adhesive, and fix them on the SEM sample stage for gold-spraying treatment. After completing the paper pattern, click Vent to release the vacuum. After waiting for a few minutes, place the sample into the sample chamber and click Pump to start vacuuming. After completion, turn on the voltage, locate the sample focus, select the appropriate area to set the magnification scale to 500 nm, and obtain the SEM image of the ATM.

The UV visible absorption peaks of ATM and PLE were tested using a UV visible spectrophotometer with model number 14004422 TU-1901. Centrifuge these two types of particles separately and dilute them with deionized water to the appropriate concentration for testing. After self-checking of the instrument, the wavelength range is selected as 350–800 nm, the metering method is Abs, the sampling interval is 1 nm, and the absorbance range is 0–1.1. Fill the quartz colorimetric dish with deionized water and click on the baseline measurement. Then, take out the colorimetric dish and place it in a dish containing deionized water solution of ATM and PLE. Click Start to obtain the UV–visible absorption peak spectra of both. The infrared spectra of ATM and FOS were tested using a Fourier-transform infrared spectrometer model 14004420 IS10. Firstly, we prepare the dry solid particles of ATM and FOS. Turn on the instrument and computer power, preheat for more than 30 min, and ensure that the light source and detector are stable. Check if the beam intensity in the software reaches 70% or above. Mix 1–2 mg of sample with 100–200 mg of dry KBr powder, grind, and press into a transparent sheet. Clean the sample compartment and place KBr blank reference. Select ’Collect Background’ in the software, set the scanning frequency and resolution, and save the background spectrum. Then, place the prepared sample into the sample compartment, and select “Collect Sample”; the parameters are consistent with the background scan, and the software will automatically deduct the background and display the absorption spectra of ATM and FOS.

To investigate the self-driving behavior of ATM under proton gradients, we used an Olympus IX71 inverted microscope to study its motion path under different concentration gradients. Turn on the computer, open cellSens (Standard 1.18) software, turn on the power of the microscope light source, and adjust the brightness of the bright field. Centrifuge the ATM and prepare a 1 mg/mL solution using distilled water. Wash the glass slide and cover slide with ethanol, dry them, and place the glass slide in the center of the bright field lamp on the observation table. Drop a drop of ATM solution onto the central part, then add a drop of distilled water with a pH of 7, and cover the cover slide. Adjust the focal length and select a suitable position for observation, and record its motion trajectory and analyze it. Replace distilled water with hydrochloric acid solutions with pH values of 6, 5, and 4, repeat the above experiment, and record the movement trajectory. After repeating the above experiment three times, the average MSD image is obtained by processing the motion trajectory. Then, according to the formula MSD = 4D_eff_Δt, the MSD images of different pH gradients in each group are linearly fitted to obtain the slope, which is MSD in the formula. Δt is 1 s, and the diffusion coefficient D_eff_ of different ΔpH groups can be calculated, and the standard deviation of each parallel group can be calculated. The position coordinates processed from the motion trajectory can be used to calculate the displacement of ATM motion for different ΔpH groups within 18 s, and the velocity can be calculated by adding the standard deviation between three parallel groups.

### 2.4. MTT and Animal Experiments

Inoculate L929 cells into a 96-well plate and culture in an environment of 37 °C and 5% CO_2_ for 24 h until they adhere to the wall. After discarding the original culture medium, PBS solutions containing 50, 100, 150, and 200 μg/mL ATM (experimental group) and equal volumes of pure PBS (control group) were added, with 5 wells in each group. Incubation was continued for 12 h. Subsequently, 20 μL of MTT solution (5 mg/mL) was added to each well and incubated at 37 °C in the dark for 4 h. After discarding the supernatant, add 150 μL DMSO and shake to dissolve the formazan crystals. Use an enzyme-linked immunosorbent assay (ELISA) reader to measure the absorbance value at 570 nm (reference wavelength 630 nm). Calculate the average and standard deviation of the relative activity of five groups of cells obtained. Use one-way ANOVA to analyze the obtained data, and then conduct Tukey’s post hoc test.

Nine female Kunming mice, with an average body weight of approximately 20 g, were divided into three groups: the control group, the dexamethasone (bioreagent, ≥97%, Aladdin, Shanghai, China) treatment group, and the dexamethasone + ATM treatment group (n = 3) (Table 1). In the latter two groups, the mice were treated via intra-peritoneal injection of a dexamethasone aqueous solution at a concentration of 0.5 mg/mL [32]. The injection dose was 5 mg/kg body weight per day, administered once daily for one week. The dose could be adjusted according to the physiological conditions of the mice. Conversely, the mice in the control group were intra-peritoneally injected with 0.9% normal saline as a control for observation. The injection volume was 10 mL/kg body weight. All three groups of mice were housed in a dark and well-ventilated environment in order to prevent excessive stimulation and startle, which could potentially interfere with the model construction. Simultaneously, it was ensured that they were provided with sufficient and continuous maintenance feed and tap water. This was crucial in order to avoid weight loss due to other factors, which might otherwise affect the construction of the muscular wasting model. The treatment phase commenced after one week.

**Scheme 2:**
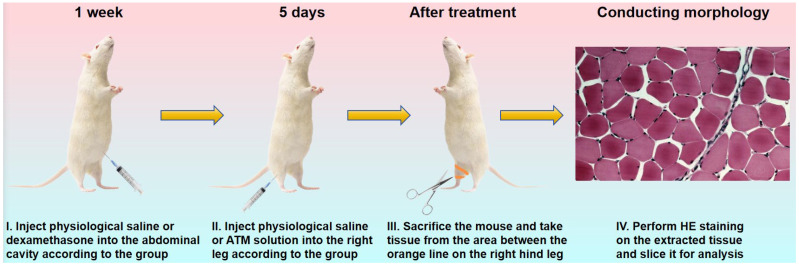
Injection site and anatomical location in the experimental procedure of each group of mice.

Following the treatment of the above-mentioned three groups of mice with the corresponding normal saline and dexamethasone solutions, the mice in the dexamethasone + ATM treatment group were treated with an ATM aqueous solution by intramuscular injection. The concentration of the ATM solution was 150 μg/mL, and the injection dose was 0.05 mL, administered into the right leg. For the other two groups (the control group and the dexamethasone-only treatment group), the same volume (0.05 mL) of 0.9% normal saline was injected into the right leg. The injection treatment for all three groups of mice was carried out once daily for 5 days. During this period, the average daily weight with standard deviation and its changes of the mice in the three groups were carefully recorded. Subsequently, the differences between the three experimental groups were analyzed. At the conclusion of the treatment, the mice were sacrificed. The thigh and calf muscles of their right lower limbs were processed into HE-stained sections (Scheme 2). Using an inverted optical microscope model Olympus IX71, select an objective lens with a magnification of 20x to observe stained sections of leg muscle tissue from three experimental groups of mice. The tissue morphology differences among the three experimental groups were compared and analyzed in detail. This included aspects such as the shape, size, and dispersion of cells, as well as the gap between muscle fibers.

All animal experiments were conducted in accordance with the 3R principles (Replacement, Reduction, Refinement) and the guidelines for the care and use of experimental animals of Northeast Forestry University, and approved by the Animal Ethics Committee of Northeast Forestry University (Approval number: 2022040. Approval Date: 7 April 2022).

## 3. Results and Discussion

**Figure 1:**
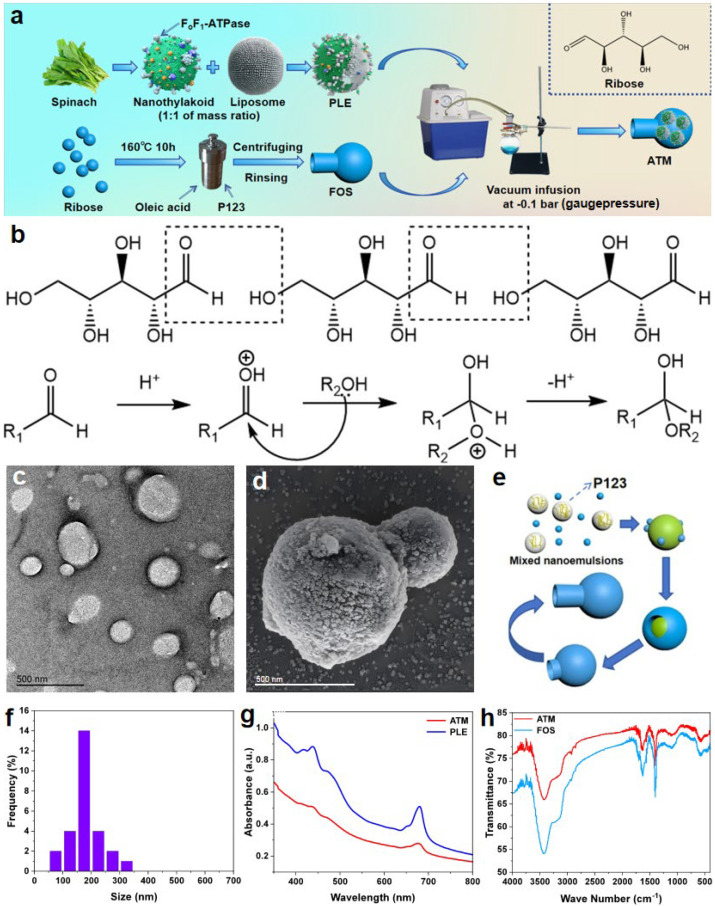
(**a**) Schematic image of synthesis and preparation of ATM. (**b**) Mechanism of aldol polymerization of D-ribose. (**c**) TEM image of PLE. (**d**) SEM image of ATM. (**e**) Formation mechanism of flask-like shape (FOS). (**f**) Particle size statistical graph of PLE. (**g**) UV/Vis spectrum of PLE and ATM in water. (**h**) Fourier infrared spectra of dried FOS and ATM.

Utilizing the method depicted in Figure 1a, a nanomotor ATM around 1 μm in size was synthesized. This nanomotor features a rotatable ATP synthetase within a flask-like structure. Spinach, being a relatively inexpensive vegetable, contains natural enzymes that serve as highly efficient ATP-synthesis tools, which holds the promise of enabling the low-cost and high-yield production of the drug. Utilizing the relevant modified methods above, it has been confirmed that the nanothylakoids extracted from spinach consist of a phospholipid bilayer, along with pigments like chlorophyll and proteins (such as F_o_F_1_-ATPase, which is essential for ATP synthesis) that are either embedded or attached to it [22,33]. However, under a vacuum condition of −0.1 bar (gauge pressure), it is rather challenging for native thylakoid vesicles to penetrate the narrow channels of the FOS. Consequently, when we blend it with lecithin lipids in the same mass ratio, we can significantly enhance its flexibility and deformability [22]. This ensures that it can smoothly enter the FOS during vacuum injection. The transmission electron microscope image of PLE (Figure 1c) reveals that PLE is a relatively dispersed closed liposome. The average particle size of most of the particles is approximately 150 to 170 nm (Figure 1f). Although there are some particles with slightly larger or smaller sizes, their statistical frequency is low. Moreover, there are no particles with particle size values exceeding 400 nm, which, to a certain degree, alleviates the technical difficulties associated with the vacuum injection of PLE into FOS, thereby ensuring the high-efficiency synthesis of ATM.

For the synthesis of FOS, the hydrothermal reaction involves several main stages (Figure 1e). Initially, at 30 °C, the template agent P123 copolymer is mixed with oleic acid and ribose in an aqueous solution. This leads to the formation of spherical micelles. The P123 copolymer consists mainly of a polypropylene oxide (PPO) core and an outer layer of polyethylene oxide (PEO). In the inner layer of the micelles, PEO interacts with oleic acid, thereby immobilizing the stencil. Meanwhile, PPO serves as a soft template for ribose polymerization, guiding ribose molecules to aggregate on the micelles. As the temperature rises, the hydrophobicity of the PEO segments of P123 gradually increases, which causes the spherical micelles to expand. With a further temperature increase, ribose undergoes additional polymerization (Figure 1b) on the micelle surface. This polymerization hinders the further aggregation of the micelles. The hydrophobic bulging of the PEO micelles further elevates their surface tension. Once this tension reaches a specific threshold, it causes the pentose shell to break outward to a certain degree. At this point, due to the inherent hydrophobicity of the ribose intermediates, they spontaneously migrate from the hydrothermal solution towards the oleic-acid center. Subsequently, the P123 template agent within is expelled, which enables the formation of a new soft template at the fracture site. As a result, ribose can continue to polymerize and extend at the fracture site, ultimately forming the bottleneck part of the FOS and continuing until a flask-shaped shell is polymerized. Given that RNA contains D-ribose and it is an essential component of ATP, we postulate that this ribose-based shell is relatively less prone to evoking immune rejection in organisms. As depicted in the scanning electron microscope image (Figure 1d) of ATM, the overall length of the ATM particle (including the neck and the spherical bottom) is approximately 1 μm. The outer diameter of the neck is around 400 nm, and that of the spherical bottom is about 600 nm. These results indicate that the width of the bottle-mouth is sufficient to ensure that most of the PLE, with a particle size of approximately 150–170 nm, can smoothly enter the FOS via vacuum-extrusion injection, enabling the effective synthesis of ATM.

To obtain a self-driven nanomotor ATM capable of synthesizing ATP, the dried FOS particles are dispersed and suspended in a buffer solution containing PLE. This mixture is then transferred to a round-bottom flask, which is connected to the extraction line of a vacuum pump via an interface. At this stage, the air inside the FOS is pumped out, enabling the injection of PLE into it to form the ATM. After being obtained through centrifugation and washing, ATM must be stored in an ADP buffer at pH 7.8 due to the fact that the ADP in the solution can supply the raw material for the motor’s ATP synthesis. Additionally, it helps simulate the original biological environment of F_o_F_1_-ATPase, preventing the loss of its activity due to its detachment from chloroplasts. Moreover, if the buffer is acidic, ATM may synthesize ATP in the buffer prematurely, which would undermine the rigor of subsequent animal experiments. In this study, the acidic characteristics of atrophic muscle tissue are utilized to verify that ATM can be driven in vivo to synthesize ATP for treating muscle atrophy symptoms in living mice. If it cannot be ensured that the initially injected ATM solution contains almost no ATP, it becomes impossible to affirm that ATM synthesizes ATP in vivo, thus affecting the experimental accuracy. As can be observed from the scanning electron microscope image (Figure 1d) and profile images of FOS and ATM (Figure S1) under an optical microscope, the ATM retains the original flask-like morphology of the FOS, along with the corresponding body length, outer diameter of the bottle-mouth, and outer diameter of the spherical bottom. This indicates that the −0.1 bar (gauge pressure) vacuum environment does not cause a significant squeezing or deformation of the FOS shape. Moreover, the FOS does not exhibit an obvious expansion after being filled with PLE, ensuring the stability and efficiency of its motion performance. In the UV–Vis absorption spectra of PLE and ATM (Figure 1g), we discovered that PLE and ATM have the same absorption peaks at 439 nm and 680 nm, indicating that PLE has been loaded inside ATM. An infrared spectroscopic analysis (Figure 1h) revealed characteristic absorption bands for both FOS and ATM. The prominent peaks observed at 3410 cm⁻^1^, 1620 cm⁻^1^, 1400 cm⁻^1^, and 1100 cm⁻^1^ correspond to the following: (1) the O-H stretching vibrations of secondary alcohols, (2) the C=O stretching of aldehyde groups, (3) the in-plane bending of primary alcohol O-H bonds, and (4) the C-O-C asymmetric stretching in ether linkages, respectively. These spectral features confirm the occurrence of condensation reactions between aldehyde and hydroxyl groups during the hydrothermal process, leading to ether bond formation while preserving the secondary alcohol functionalities of ribose. To assess the colloidal stability of ATM, zeta potential measurements were performed under both aqueous and physiological conditions. As shown in Figure S2, ATM exhibited a zeta potential of −20.5 ± 1.6 mV in distilled water, indicative of ATM’s certain degree of colloidal stability, and is relatively dispersed in this medium. In PBS solution, the zeta potential decreased to −10.9 ± 0.6 mV due to the charge shielding by physiological ions, yet remained within the range (−10 to −30 mV) associated with stable colloidal dispersions. These results confirm that ATM maintains sufficient repulsive forces to prevent aggregation during storage and under simulated biological conditions.

**Figure 2:**
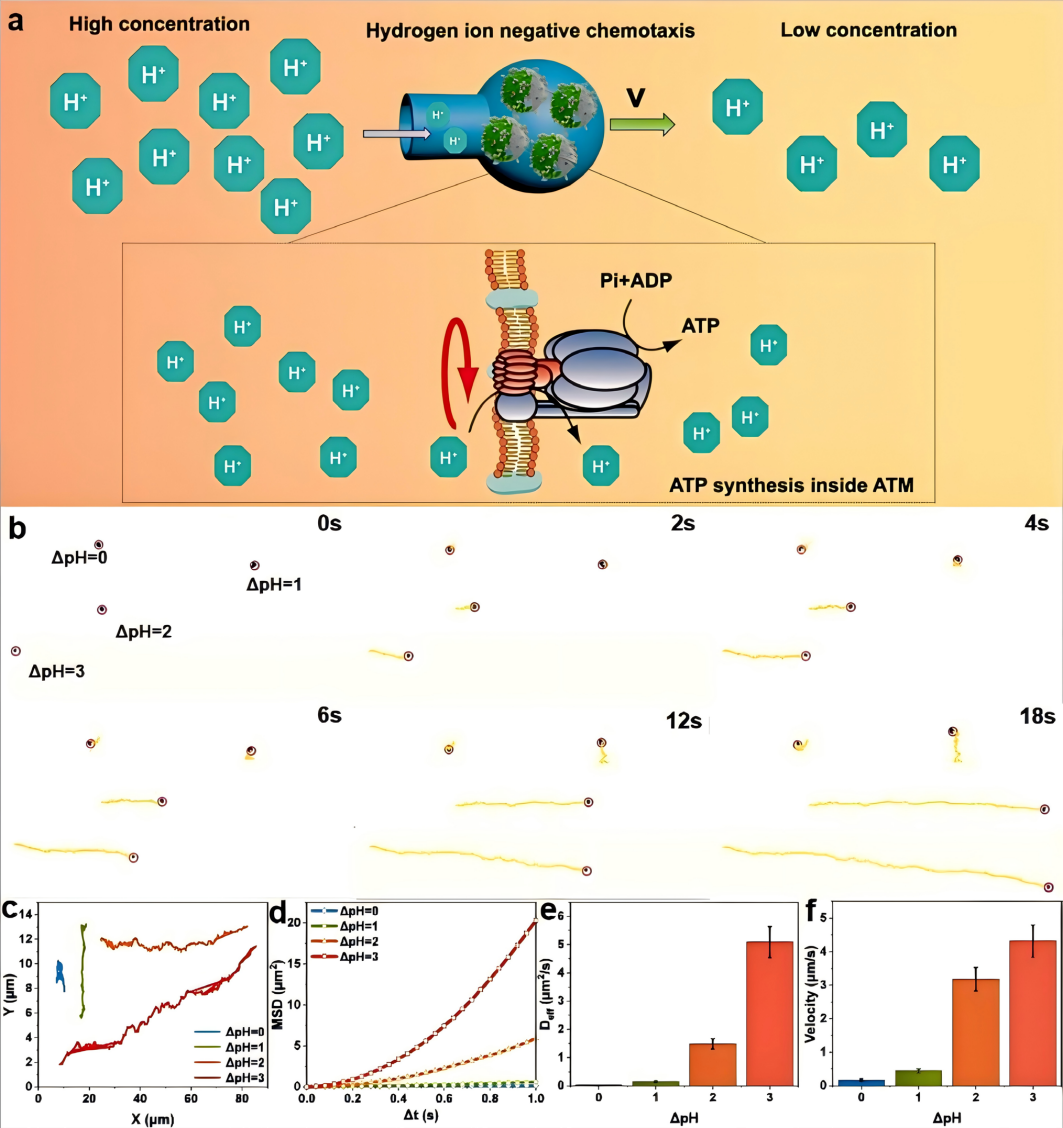
(**a**) Mechanism of endogenous driving of ATM induced by hydrogen ion concentration gradient. (**b**) The motion state of ATM at 0, 2, 4, 6, 12, and 18 s under different ΔpH conditions. (**c**) The trajectory of ATM at different pH environment in 18 s. (**d**) The MSD analysis of ATM with different ΔpH values in 1 s, which corresponds to its trajectory (**c**). (**e**) The effective diffusion coefficient D_eff_ of ATM calculated from its MSD (**d**). (**f**) The endogenous driving velocity of ATM at different pH gradients.

In the subsequent work, we carried out a comprehensive study on the spontaneous negative chemotaxis-a directional movement away from higher concentrations of a chemical stimulus’ (in this case, hydrogen ions) behavior of the endogenous driven nanomotor ATM. This behavior is induced by different hydrogen-ion concentration gradients (pH differences) in an aqueous solution. In an environment with a hydrogen-ion gradient, ATM spontaneously exhibits negative chemotaxis, moving from the region of a high hydrogen-ion concentration to that of a low concentration. It achieves so with its spherical bottom leading and the bottleneck opening trailing, presenting a fluid-flow model characterized by a wide front and a narrow back. This configuration can significantly enhance its directional stability. Simultaneously, relatively high-concentration hydrogen ions can enter the ATM through the bottleneck opening. Once inside, they utilize the F_o_F_1_-ATPase on PLE to convert chemical potential energy into energy stored within ATP molecules (Figure 2a). Theoretically, both ADP and Pi can be utilized both inside and outside the PLE phospholipid membrane to synthesize ATP. This synthesized ATP can be stored inside the ATM and later used for treating related diseases. In this study, we employed an Olympus IX71 inverted microscope to stimulate the self-driving behavior of ATM. We used distilled water and hydrochloric acid solutions with pH values of 7, 6, 5, and 4, respectively. First, we dropped a 1 mg/mL ATM solution in the center of a glass slide. Subsequently, we added the distilled water and hydrochloric acid solutions with pH values of 6, 5, and 4, respectively, and then carefully observed and recorded their movement behavior and trajectory.

After processing the trajectory using ImageJ software, it was found that the endogenously driven ATP synthesis motor ATM exhibited significant motion behavior in the presence of hydrogen ion concentration differences, which is distinctly different from the Brownian motion of FOS and ATM in a neutral aqueous solution (Movies S1 and S2). From the screenshots of the motion video (Figure 2b and Movie S3), apart from the group with ΔpH = 0, all other groups of ATM motor particles exhibited varying degrees of movement, which accurately validates the driving effect of the hydrogen-ion gradient on ATM. Furthermore, we can observe that, for the two groups with ΔpH = 2 and ΔpH = 3, which are moving in essentially the same direction, at time 0, the position of the latter lags significantly behind that of the former. However, as time elapses, the motor particles in the ΔpH = 3 group gradually catch up with those in the ΔpH = 2 group and, ultimately, reach the same position by 18 s. This indicates that the speed of the ΔpH = 3 group is significantly higher than that of the ΔpH = 2 group, demonstrating that different hydrogen-ion gradients have distinct impacts on the motion speed. The average distances of motion of ATM at ΔpH values of 0, 1, 2, and 3 are approximately 2.83 μm, 7.84 μm, 57.21 μm, and 77.67 μm, respectively (Figure 2c). This shows that ATM has significantly different endogenous driving capabilities in different proton-gradient environments. Notably, when ΔpH is 2, its motion ability is significantly enhanced compared to when ΔpH = 1. In addition, the diffusion capability of ATM can be characterized by the azimuthal shift, and its formula is MSD = 4D_eff_Δt. In this equation, MSD stands for the mean square displacement, D_eff_ represents the effective diffusion coefficient, and Δt denotes the time interval. In the MSD curve of ATM (Figure 2d), when ΔpH = 0, the MSD function exhibits a linear increase with time. This linearity is characteristic of the fundamental Brownian motion. As the value of ΔpH increases, the MSD curve gradually takes on a parabolic shape, which is in accordance with the aforementioned equation. Specifically, the MSD curve at ΔpH = 2 demonstrates a substantial improvement in comparison to the curve at ΔpH = 0. Moreover, when the ΔpH is increased beyond this proton gradient, the growth rate of the MSD curve will be further enhanced. This indicates that the change in the hydrogen-ion concentration gradient, represented by ΔpH, has a significant impact on the diffusion behavior of ATM, as reflected by the MSD curve. The more pronounced the proton gradient, the more remarkable the deviation from the simple Brownian motion and the greater the enhancement in the diffusion-related characteristics of the nanomotor ATM.

Utilizing the formula mentioned above, we are able to compute the effective diffusion coefficient D_eff_ of ATM. The D_eff_ values corresponding to pH levels of 7, 6, 5, and 4 are 0.02 ± 0.005 μm^2^/s, 0.14 ± 0.02 μm^2^/s, 1.48 ± 0.18 μm^2^/s, and 5.09 ± 0.55 μm^2^/s, respectively (Figure 2e). These data further validate the negative chemotactic behavior of ATM in relation to hydrogen ions across different pH environments. Through the analysis of its motion trajectory within an 18 s time frame, we can determine its average driving speed in an aqueous solution environment. This is achieved by connecting the starting and ending points of the motion trajectory, calculating the length of this line segment, and then dividing it by the 18 s time interval. Post-calculation, we discovered that the average velocities of ATM in solution environments with ΔpH values of 0, 1, 2, and 3 are 0.16 ± 0.04 μm/s, 0.44 ± 0.06 μm/s, 3.18 ± 0.35 μm/s, and 4.32 ± 0.48 μm/s, respectively (Figure 2f). Compared to other enzyme-powered nanomotors reported recently [34], ATM demonstrates superior motility under physiological pH gradients. This enhanced performance can be attributed to the unique flask-shaped structure that facilitates directional movement through fluid dynamic optimization. This finding not only attests to the impact of the hydrogen-ion environment on ATM but also offers a reference for estimating the velocity of ATM within a biological liquid environment.

**Figure 3:**
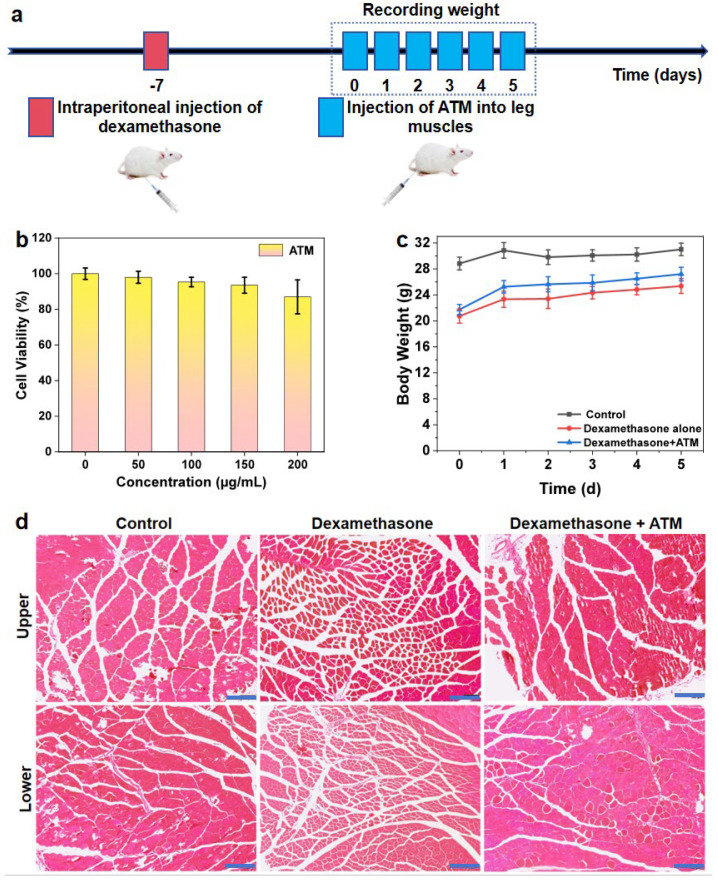
(**a**) Experimental flowchart for treating muscle atrophy in mice. (**b**) Relative viabilities of L929 cells treated with ATM (n = 5). (**c**) Body weights of mice of different experimental groups (n = 3). (**d**) H&E staining of mice right lower limb of different experimental groups (Objective: MPLN20X (20×), scale bar: 200 μm).

For this endogenous motor capable of self-driving and internal ATP storage, we can contemplate the rational utilization of the ATP stored during its motion to treat diseases such as muscle atrophy and myocardial infarction, which are caused by abnormal cellular energy metabolism. Consequently, we resolved to explore the efficacy of ATM in treating muscle atrophy symptoms in mice, aiming to provide preliminary evidence and feasibility for clinically addressing conditions like muscle weakness. Prior to the practical application of ATM for treatment, we initially examined its impact on the activity of L929 cells. We incubated L929 cells for 12 h using a PBS solution and ATM aqueous solutions with concentrations of 50 μg/mL, 100 μg/mL, 150 μg/mL, and 200 μg/mL respectively. The MTT assay revealed a concentration-dependent but statistically non-significant effect of ATM on L929 cell viability within the therapeutic dose range (Figure 3b). A quantitative analysis showed cell viabilities of 97.97 ± 3.37% (50 μg/mL), 95.32 ± 2.66% (100 μg/mL), 93.52 ± 4.46% (150 μg/mL), and 86.98 ± 9.53% (200 μg/mL) compared to PBS controls (set as 100 ± 3.19%). A statistical analysis using one-way ANOVA with Tukey’s post hoc test indicated no significant differences (*p* > 0.05) between concentrations up to 150 μg/mL, while the 200 μg/mL group showed a marginal but statistically significant decrease (*p* < 0.05). This dose–response pattern suggests that ATM maintains >90% cell viability at concentrations ≤150 μg/mL, which guided our selection of 150 μg/mL for subsequent in vivo experiments. Although the concentration of 200 μg/mL showed a 6.54-percentage-point decrease compared to the previous concentration group of 93.52% (which is greater than the differences of 2.65% and 1.80% between the first three groups), the cells still maintained a relatively high level of activity. This indicates that the material of the ATM motor exhibits high biocompatibility, and its application in the treatment of live animals holds a certain degree of feasibility. While the 12 h cytotoxicity assay demonstrates excellent biocompatibility, the long-term fate of ATM components requires further investigation. The phospholipid composition suggests potential biodegradation through endogenous lipase activity [35], and the ribose-based shell may undergo gradual hydrolysis under physiological conditions [36]. Future studies should include chronic toxicity assessments and a detailed biodistribution analysis to confirm complete metabolic clearance. Taking all factors into account, to achieve more notable therapeutic effects while sustaining high cell activity, we selected a concentration of 150 μg/mL when treating mice with ATM.

Therefore, in accordance with the determined timeline (Figure 3a), we selected nine mice with comparable weight and health conditions and divided them into three groups: the control group, the diseased group, and the treatment group. Dexamethasone can induce muscle atrophy symptoms in normal and healthy mice through various pathways. These include imbalances in cellular protein metabolism [37], the inhibition of muscle regeneration [38], mitochondrial dysfunction [39], and genetic autophagy [40]. Consequently, we utilized a dexamethasone aqueous solution at a dose of 5 mg/kg 30 to induce muscle atrophy symptoms, which mainly manifested as weight loss and a reduction in cell plumpness. After 7 days of treatment with dexamethasone, we observed that the average body weight of mice in the other two groups on day 0 of treatment was significantly lower than that of normal healthy mice (Figure 3c). The average weight difference between the diseased group and the healthy group was 8.10 g, while the treatment group had a difference of 7.09 g compared to the healthy group. This clearly indicates that dexamethasone effectively plays a role in constructing the muscle atrophy model. Following the cessation of dexamethasone treatment, we administered physiological saline to the diseased group and 150 μg/mL ATM solution to the treatment group. As a result, the average body weight of both groups of mice increased to a certain extent. Notably, the growth rate was the highest on the first day after changing the drugs, and then gradually slowed down. However, overall, the mice continued to gain weight steadily every day. On the fifth day after treatment, the difference in average body weight between the diseased group and the treatment group was more pronounced compared to the start of treatment. The difference in average body weight between them and the control group on the fifth day was 5.66 g and 3.81 g, respectively. This demonstrates that ATM can indeed accelerate the weight recovery of muscle-atrophy mice to a certain degree and widen the gap compared to untreated mice.

The selective accumulation of ATM in atrophic tissues might be attributed to two synergistic mechanisms: First, negative chemotaxis pushes ATM to the low-proton-concentration region unique to the diseased tissues [41]. Secondly, ATM can be ingested through the endocytosis or macropinocytosis of atrophic cells into them [42]. To further verify the therapeutic effect of the proton-driven nanomotor ATM on muscle atrophy, we conducted hematoxylin and eosin (H&E) staining on the upper and lower muscle tissues of the right lower limbs of all experimental groups of mice. This staining revealed the differences and connections in tissue morphology among the three groups of mice (Figure 3d). Based on the results of the stained sections, we can conclude that the muscle tissue of the control-group mice consists of numerous relatively large, block-like muscle groups. Each of these groups is composed of many tightly arranged skeletal muscle cells. The spacing between the muscle membranes of these cells is extremely narrow, and most of the cells exhibit a full, rounded-square shape, with most particle sizes ranging from 30–40 μm. In contrast to the control group, the leg tissue of the diseased-group mice showed a higher density of individual skeletal muscle cells. Although block-like muscle groups could still be faintly discerned, the cells that composed them were shorter and more angular, and the gaps between the muscle membranes were larger, resulting in a more scattered appearance. The particle size of most cells was concentrated between 15–25 μm, further validating the effectiveness of dexamethasone in inducing the disease. The upper-limb muscle tissue of the treatment-group mice was found to be similar to that of the control group, consisting of block-like muscle groups composed of tightly arranged single muscle cells. The cell particle size was also comparable to that of the control group, with a rounded and full shape. For the lower part of the tissue in this group, although there were some gaps between the cell membranes of individual cells and the surrounding muscle groups, most skeletal muscle cells still exhibited a tightly arranged and plump morphology, which was basically consistent with the tissue morphology of the corresponding parts of the control group. The cells in the treatment group were in stark contrast to those in the diseased group. The former had a large individual volume, were full, and were tightly arranged, closely resembling the control group, while the latter had short and scattered cells with distinct edges, consistent with the tissue characteristics of muscle atrophy. This confirms that ATM is expected to achieve the desired therapeutic effect in the treatment of muscle-atrophy-related diseases and holds great potential for clinical application.

Although this study demonstrates the potential of ATM in treating symptoms of muscle atrophy, there are still some limitations. Firstly, the muscle atrophy model is constructed through drug induction, and its pathological characteristics may differ from naturally occurring muscle atrophy, which limits the generalizability of the conclusions. Secondly, although ATM exhibits a high biocompatibility in in vitro experiments, the long-term in vivo immune response and metabolic clearance mechanisms remain unclear. Finally, although the driving mechanism of ATM is based on the proton gradient, its movement in complex biological microenvironments still needs to be studied.

From the perspective of clinical translation, this study provides new ideas for the treatment of metabolic diseases, but still faces many challenges. The transformation from mouse models to human applications requires systematic validation, including dose optimization, administration routes, and safety evaluation. The large-scale production and quality control of ATM are key to achieving clinical applications, and standardized preparation processes need to be developed to ensure batch consistency. Future research can explore the synergistic effects of ATM with other therapies such as gene therapy or physical rehabilitation [43] to enhance overall efficacy. Moreover, it is necessary that we thoroughly evaluate the immunogenicity, long-term toxicity, and targeting of ATM in the human body, laying the foundation for its entry into clinical trials. Despite the challenges, ATM has demonstrated broad application prospects in the field of precision medicine due to its endogenous drive and high biocompatibility.

## 4. Conclusions

Overall, we have successfully demonstrated the potential of a flask-shaped F_o_F_1_-ATPase nanomotor. This nanomotor can perform endogenous negative chemotaxis within a hydrogen-ion environment. Simultaneously, it is capable of synthesizing and storing ATP, which shows promise in treating muscle atrophy symptoms. ATM can ingeniously capitalize on the acidic microenvironment of atrophied tissues. It converts the chemical potential energy of proton-concentration gradients into energy stored within ATP molecules. By doing so, it promotes ATP synthesis and enhances the metabolism of atrophied cells. Furthermore, ATM displays distinct movement behaviors across different hydrogen-ion environments. It possesses a relatively rapid self-driving ability, guaranteeing high efficiency during the treatment process. The material of ATM exhibits high biocompatibility. This property provides the immunological basis for exploring its applications in the biological and medical fields. This type of nanomotor makes use of the organic assembly of natural enzymes and chemically synthesized materials. It has shown promising therapeutic effects in inhibiting muscle atrophy symptoms in mice. As a result, it offers a novel approach to drug design for treating related metabolic disorders.

## Figures and Tables

**Table 1:** Drug treatment methods and setting objectives for each group of mice.

Group	Number of Mice	Intraperitoneal Injection (Daily, 1 week)	Right Leg Muscle Injection (Daily, 5 days)	Purpose
Control Group	n = 3	0.9% saline (10 mL/kg)	0.9% saline (0.05 mL)	Baseline comparison for muscle morphology and weight changes.
Dexamethasone (DEX) Group	n = 3	Dexamethasone (5 mg/kg; 0.5 mg/mL solution)	0.9% saline (0.05 mL)	Induce muscle wasting via glucocorticoid treatment.
DEX + ATM Group	n = 3	Dexamethasone (5 mg/kg; 0.5 mg/mL solution)	ATM solution (150 µg/mL; 0.05 mL)	Evaluate ATM’s protective/therapeutic effects against dexamethasone-induced wasting.

## Data Availability

The data generated and analyzed during this study are available from the corresponding author upon request. The data are not publicly available due to privacy restrictions involving research participants.

## Supplementary Materials

The following supporting information can be downloaded at: https://www.mdpi.com/article/10.3390/ma18061351/s1, Figure S1: Profile images of FOS (a) and ATM (b) under an optical microscope; Figure S2; Zeta potential of ATM in distilled water (−20.5 ± 1.6 mV) and PBS solution (−10.9 ± 0.6 mV) respectively; Movie S1: Brownian motion of FOS; Movie S2: Brownian motion of ATM; Movie S3: Motion behavior of ATM under different ΔpH conditions.
