# Supplementary material for: An Endogenous Proton-Powered Adaptive Nanomotor for Treating Muscle Atrophy
Source: Materials (Basel). 2025 Mar 19;18(6):1351. doi: 10.3390/ma18061351 (PMC11943966; doi:10.3390/ma18061351)
Supplement: Supplementary file 1 [file materials-18-01351-s001.zip › materials-3505609-supplementary.pdf]

Supplemental Materials for:

An Endogenous Proton-Powered Adaptive Nanomotor for Treating Muscle Atrophy

Ming Liu †, Zhicun Liu †, Xiangkai Qiao †, Cheng Chen , Hongtu Guo , Hao Gu *, Junbo Li * and Tiedong Sun *

College of Chemistry, Chemical Engineering and Resource Utilization, Northeast Forestry University, Harbin 150040, China; 6ming@nefu.edu.cn (M.L.); 2022214670@nefu.edu.cn (Z.L.); guoht@nefu.edu.cn (H.G.)

* Correspondence: guhao@nefu.edu.cn (H.G.); lijunbo91@nefu.edu.cn (J.L.); tiedongsun@nefu.edu.cn (T.S.)

† These authors contributed equally to this work.

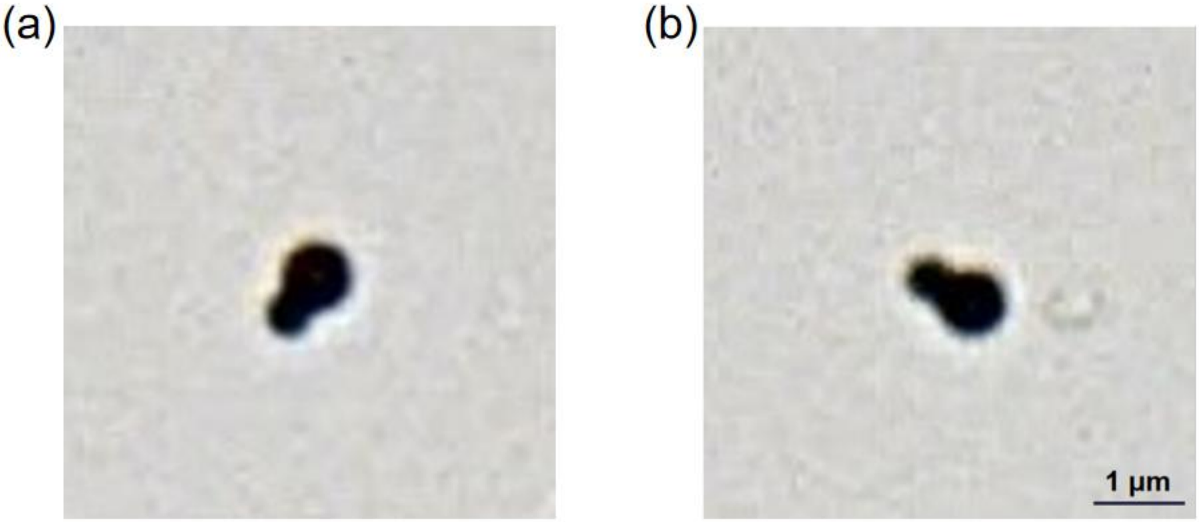

Figure S1. Profile images of FOS (a) and ATM (b) under an optical microscope.

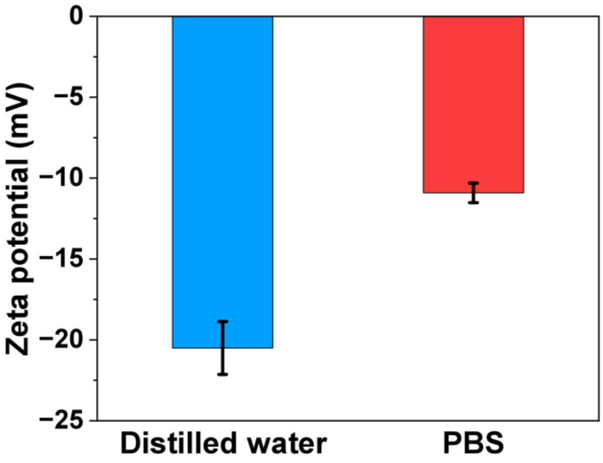

Figure S2. Zeta potential of ATM in distilled water (-20.5 ± 1.6 mV) and PBS solution (-10.9 ± 0.6 mV) respectively
